# Comparative Study of Oral Health-Related Quality of Life (OHRQL) between Different Types of Orthodontic Treatment

**DOI:** 10.3390/medicina57070683

**Published:** 2021-07-02

**Authors:** Natalia Zamora-Martínez, Vanessa Paredes-Gallardo, Verónica García-Sanz, José Luis Gandía-Franco, Beatriz Tarazona-Álvarez

**Affiliations:** Department of Orthodontics, Dentistry School, University of Valencia, 46010 Valencia, Spain; natalia.zamora@uv.es (N.Z.-M.); vanessa.paredes@uv.es (V.P.-G.); jose.l.gandia@uv.es (J.L.G.-F.); beatriz.tarazona@uv.es (B.T.-Á.)

**Keywords:** oral health-related quality of life, orthodontic treatment needs, malocclusion, patient assessment

## Abstract

*Background and objectives:* Although the main objective of any orthodontic treatment is to correct malocclusion, a range of psychosocial and/or esthetic factors drive patients to undergo orthodontic treatment. The aim of the present study was to analyze variations in oral health-related quality of life (OHRQL) levels in patients undergoing orthodontic treatment by means of four types of appliances: fixed buccal metal brackets, fixed buccal esthetic/ceramic brackets, fixed lingual brackets, and clear aligners. *Material and Methods*: The study sample comprised 120 patients aged 18 to 68 years who attended the Orthodontic department at the Dental Clinic of the University of Valencia. The Index of Orthodontic Treatment Need (IOTN) was used to measure orthodontic treatment need. Each patient completed three different intervals of the 14-item Oral Health Impact Profile (OHIP-14): before treatment (T0); six months after placing the orthodontic appliances (T1) and at the end of orthodontic treatment (T2). *Results*: All groups suffered a reduction in quality of life from T0 to T1 except the metal bracket group which presented the same level for the functional limitation domain (*p* = 1.000), the lingual bracket group for the psychological discomfort domain (*p* = 1.000) and clear aligner group for the physical disability domain (*p* = 0.118) and psychological disability domain (*p* = 1.000). Nevertheless, quality of life for most domains was similar in all groups at the end of treatment (T2). *Conclusions*: Patients underwent a significant reduction in quality of life during treatment in comparison with their pre-treatment condition but showed significant improvements at the end of treatment.

## 1. Introduction

The main objective of any orthodontic treatment is to correct malocclusion. However, it is often not the malocclusion but a range of psychosocial and/or esthetic factors that drive patients to undergo treatment. The improvements in function and esthetics resulting from orthodontic treatment constitute an improvement in the patient’s wellbeing and quality of life [1]. A wide range of orthodontic appliances are available and the choice depends on both the type of malocclusion, the existence of associated disorders and the patients’ esthetic and functional demand [2,3,4].

Health-related quality of life has been associated to many factors, individual and environmental, which suggests that gender plays an important role in this regard [5,6,7]. Oral health-related quality of life (OHRQL) indicators help the clinician evaluate patients’ needs and expectations, and so inform the decisions taken in treatment planning in response to the individual’s concerns [8]. Various instruments have been created to measure OHRQL levels. One of these is the Oral Health Impact Profile (OHIP), which evaluates the self-perceived dysfunction, discomfort and disability deriving from oral pathology. The original version was the OHIP-49, which has spawned various shorter versions adapted to different research needs and objectives, including the OHIP-14, a simplified version that is widely used in research for its brevity and ease of use [6].

The literature includes several studies that have analyzed OHRQL indicators in orthodontic treatment. Most of them set out to determine the level of improvement after orthodontic treatment [9,10,11,12], while a few, such as Healey et al. (2016), have assessed OHRQL in the longer term, evaluating quality of life 21 months after treatment rather than immediately following the end of treatment [13]. Many authors, including Seehra et al. [14], concluded that the main improvements obtained are in the emotional and social aspects of OHRQL.

Regarding malocclusions, Zheng et al. [6] reported that although improvements in quality of life were observed, they differed depending on the type of treatment received. In this way, patients treated for Class II presented greater changes in levels of psychological discomfort and psychological disability during the space closure phase, while Class I patients experienced greater changes during the initial phase. However, authors such as Benson et al. [7] did not find statistically significant differences deriving from the type of orthodontic treatment undertaken.

The objective of the present study was to analyze variations in oral health-related quality of life (OHRQL) levels in patients undergoing orthodontic treatment by means of four types of appliances: fixed buccal metal brackets, fixed buccal esthetic/ceramic brackets, fixed lingual brackets, and clear aligners.

## 2. Materials and Methods

A total of 120 patients were elected from among those attending the Orthodontic Clinic at the University of Valencia (Spain) seeking orthodontic treatment. Thirty consecutive patients that were previously assigned to each appliance group and met inclusion criteria were selected to participate. The assignation to the different groups depended on malocclusion and patients’ demands.

The study protocol was approved by University of Valencia Ethics Committee for Human Research (reg. code 1337124). Patients gave their informed consent to take part in the study. All were examined at the first visit to the clinic and were invited to participate in the study according to the following inclusion and exclusion criteria:

### 2.1. Inclusion Criteria

- Adult patients aged over 18 years.
- Patients presenting good oral health (without caries or periodontal disease).
- Patients presenting good general health.

### 2.2. Exclusion Criteria

- Patients who were to undergo orthognathic surgery.
- Patients who had undergone previous orthodontic treatment.
- Patients who missed more than three appointments.
- Patients presenting systemic disease.
- Incomplete protocol due to a lack of patient collaboration: (1) failure to follow the treatment regimen; (2) forms completed incorrectly.
- Patients unwilling to take part in the study.

### 2.3. Study Design

Study protocol design included estimations of sample size and statistical power. A study by Chen et al. reports a mean total OHIP-14 score of 9.06 ± 6.76 in a patient subgroup presenting a threshold need for orthodontic treatment (IOTN = 3) [11]. This deviation was used as the basis for estimations made in the present study.

It was determined that a minimum sample of 30 patients per group was required to detect a significant effect size of f = 0.25 (mean) with a power of 80% for differences between groups at a specific study time, and 99% for changes over the entire study period, assuming a confidence interval of 95%. This effect size was compatible with mean total OHIP-14 scores of 9, 10.5, 12, and 13.5 points.

Thirty patients per group were selected according to the inclusion and exclusion criteria to create four groups: buccal metal orthodontic brackets (M); buccal esthetic/ceramic brackets (E); lingual brackets (L), and clear aligners (C).

All patients completed a form recording the following data: sex, age, race, marital status, educational level. Each patient was assigned a number, so that the questionnaires remained confidential. Patients were also examined to establish the Index of Orthodontic Treatment Need (IOTN) in each case.

OHRQL indicators were analyzed using the OHIP-14 which has been shown to be a reliable and valid instrument for measuring quality of life [10,11], a questionnaire structured as seven domains: functional limitation, physical pain, psychological discomfort, physical disability, psychological disability, social disability, and handicap. Each item is scored by means of a five-point LIKERT quantifying the frequency of events impacting on quality of life for each domain, with scores ranging from “never = 0” to “always = 4” [15].

Patients completed the OHIP-14 at three study times:

- (T0): Before the start of orthodontic treatment.
- (T1): Six months after the start of orthodontic treatment.
- (T2): At the end of orthodontic treatment.

### 2.4. Statistical Analysis

Descriptive statistics were calculated: mean, standard deviation, range, and median values of the indirect variables generated by the OHIP-14, and of the partial components (continuous) and relative and absolute frequencies for individual OHIP-14 questions (categorical).

The Kolmogorov–Smirnov test was applied to check for normal distribution of total OHIP-14 scores in each group, obtaining negative results (*p* < 0.05) in most cases; for this reason non-parametric analysis was performed.

Brunner–Langer non-parametric models were estimated for longitudinal data of the main outcome (total OHIP-14 score) and each of the seven OHIP-14 domains. ANOVA-type statistics (ATS) were calculated to evaluate changes in scores over the study times according to treatment group.

Comparisons between groups at each study time were made with the Kruskal–Wallis test and multiple comparisons between pairs of groups applying the Mann–Whitney test with Bonferroni correction.

To evaluate homogeneity of the groups regarding patient classification variables, the Chi-square independence test and the Kruskal–Wallis test for independent samples were applied.

Multiple regression models using generalized estimation equations (GEE) were conducted to analyze the outcome total OHIP-14 according to group, time, and patient’s individual variables. Adjusted beta coefficients and 95% confidence intervals were obtained from the Wald’s Chi2 statistic. This approach was used because of the within-subject correlation associated to repeated measurements.

The significance level applied in statistical analysis was 5% (α = 0.05).

## 3. Results

The total sample included 120 patients, 61 men (50.8%) and 59 women (49.2%), with a mean age of 37.4 ± 14.6 years ranging between 18 and 68 years. Mean total treatment duration was 19.6 ± 4.7 months.

Regarding socio-demographic variables and the severity of the malocclusions, most of the patients were Caucasians (98.3%); two thirds (67.5%) had completed higher education, and more were single (55.8%) than married (44.2%); patients’ level of need for treatment was evaluated with the IOTN: 8.3% of patients presented grade 1, 24.2% grade 2, 30% grade 3, 25% grade 4, and 12.5% grade 5. These findings confirmed that the patient groups were homogenous whereby all four groups presented similar characteristics including the severity of their malocclusions (*p* = 0.407).

### 3.1. Evolution of Total OHIP-14 Scores

Firstly, the homogeneity of OHIP-14 results at the pre-treatment evaluation (T0) was analyzed, finding statistically significant differences in quality of life between the four study groups, as shown in Table 1.

**Figure 1:**
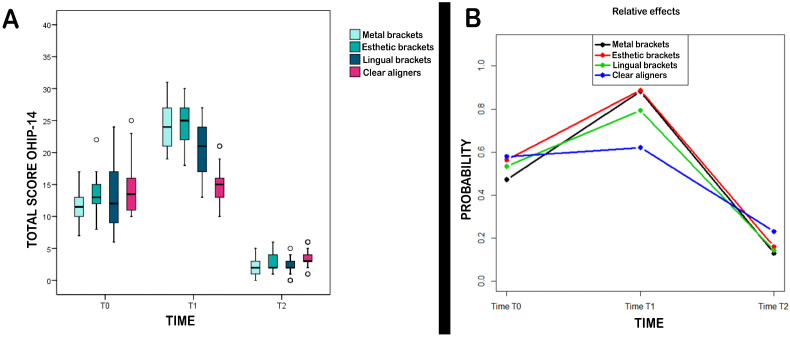
(**A**) Comparison between four types of orthodontic treatment at three study times (T0, T1, and T2); (**B**) Relative effects of groups at different study times to enable interpretation of *p*-values calculated.

Secondly, the four types of orthodontic treatment were compared at the three study times (T0, T1, and T2). The results are shown in Table 2 and Figure 1.

Regarding comparisons between groups at each study time (Figure 1A,B), significant differences were found at T0 (*p* = 0.013). Six months after the start of treatment, no significant differences were observed between the two buccal bracket groups (M and E), although they showed a significantly worse quality of life than the other two treatment types (L and C).

All groups underwent significant changes over the entire study period (Table 2) (*p* < 0.001). In this way, the Brunner–Langer model detected a significant interrelation between study time and group (*p* < 0.001), whereby total OHIP scores changed significantly over time but in different ways, depending on group.

### 3.2. Evolution of Individual OHIP-14 Domains

Comparisons of the OHIP-14’s seven domains (or partial components) at T0 are shown in Table 3. All variables presented statistically significant differences between groups.

**Figure 2:**
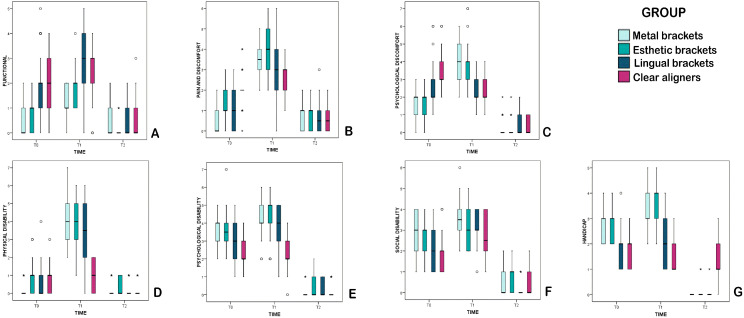
Results for each of the seven OHIP-14 domains evaluated for each type of orthodontic treatment ((**A**)**:** functional limitation; (**B**): physical pain; (**C**): psychological discomfort; (**D**): physical disability; (**E**): psychological disability; (**F**): social disability; (**G**): handicap). ° atypical values; * extreme values.

The results of comparisons between groups at each study time for each of the seven domains are shown in Figure 2. The overall evolution of the seven domains over time was significantly different between groups (*p* < 0.001, interaction).

#### 3.2.1. Functional Limitation

As shown in Figure 2, all groups underwent a reduction in quality of life in terms of functional limitation from T0 to T1 but after the end of treatment (T2), all presented improvements in comparison with T0, with the exception of the metal bracket group (M) which presented the same level as at the pre-treatment study time (T0) (*p* = 1.000). At the end of treatment, a very similar quality of life was observed in all groups, although only just (*p* = 0.096) as the bracket groups showed a slight tendency towards better outcomes.

#### 3.2.2. Physical Pain

Results for this domain (Figure 2B) presented significant differences between groups at the pre-treatment evaluation (T0), particularly between the metal brackets group (M) and clear aligners (C). Nevertheless, all groups suffered a reduction in quality of life from T0 to T1. After 6 months of treatment (T1), the esthetic/ceramic brackets group (E) showed a worse quality of life than the lingual bracket group, with notable difference in comparison with the clear aligner (C) group (*p* = 0.006). At the end of treatment, a very similar quality of life was observed in all groups. Nevertheless, only the metal brackets group (M) and the clear aligner (C) group showed significant improvements at T2 compared with T0. The esthetic/ceramic bracket group (E) showed a strong tendency towards improvement (*p* = 0.063), but quality of life in the lingual bracket group remained unchanged (*p* = 0.335).

#### 3.2.3. Psychological Discomfort

For this domain (Figure 2C), all groups except the lingual bracket group (*p* = 1.000) experienced significant reductions in quality of life from T0 to T1. At the same time, all groups showed improvements in quality of life from T0 to T2. At the pre-treatment evaluation (T0), the clear aligner group (C) presented a worse quality of life than the other three groups. After six months of treatment (T1), the buccal brackets groups (M and E) obtained a worse quality of life than the other two groups (L and C). Nevertheless, at the end of treatment (T2), quality of life was very similar in all groups.

#### 3.2.4. Physical Disability

Regarding the physical disability domain (Figure 2D), all groups except for the clear aligner group (C) (*p* = 0.118) suffered a significant reduction in quality of life from T0 to T1. At the pre-treatment assessment (T0), the metal bracket group obtained the best quality of life but after 6 months of treatment (T1), the clear aligner group (C) obtained a significantly better quality of life than the other groups. At the end of treatment (T2), quality of life was similar in all groups.

#### 3.2.5. Psychological Disability

For this domain (Figure 2E), all groups except for the clear aligner group (C) (*p* = 1.000) suffered significant reductions in quality of life from T0 to T1. As in the domains analyzed above, all groups underwent significant improvements in quality of life from the pre-treatment (T0) to the post-treatment assessment (T2). At the pre-treatment evaluation (T0), the clear aligner group (C) presented a better quality of life than the other groups. After 6 months of treatment (T1), the clear aligner group (C) maintained and improved its better quality of life compared with the lingual bracket group. Nevertheless, quality of life for this domain was similar in all groups at the end of treatment (T2).

#### 3.2.6. Social Disability

The overall evolution for this domain (Figure 2F) was almost the same in all groups (*p* = 0.020 interaction). However, the changes in quality of life were smaller than in the other domains described above. All groups suffered a reduction in quality of life from T0 to T1 and all obtained improvement from pre-treatment (T0) to post-treatment (T2). At the initial assessment (T0), the clear aligner group (C) presented a better quality of life than the three bracket groups. However, after 6 months of treatment (T1), evolution showed some homogeneity, whereby the clear aligner group maintained the better quality of life obtained at T0. At the end of treatment (T2), the lingual orthodontics group (L) obtained a significantly better score than the esthetic/ceramic brackets group (E).

#### 3.2.7. General Handicap

Lastly, OHIP-14 results for the general handicap domain (Figure 2G) showed that the buccal brackets groups (M and E) suffered significant reductions in quality of life from T0 to T1. However, the clear aligner group (C) experienced an improvement from T0 to T1, while the lingual bracket group (L) underwent no change. As in the other domains, all groups obtained significant improvements in quality of life from pre-treatment (T0) to post-treatment (T2). At the initial evaluation (T0), patients in the buccal bracket groups (M and E) obtained a significantly poorer quality of life than the lingual bracket (L) or clear aligner (C) groups. After 6 months of treatment (T1), the clear aligner group (C) obtained better results than the other three groups (M, E and L). At the end of treatment (T2), the clear aligner group (C) remained stable, with little change, while the other three groups (M, E and L) obtained much better results.

### 3.3. Gender Differences

Table 4 shows the differences in the seven domains and the total scores for the three study times between males and females. There are only some isolated significant differences. For the physical pain and physical disability domains, females show significantly higher scores than males.

### 3.4. Effects of Individual Variables

Patient-related variables (gender, age, marital status, educational level, and IOTN) were analyzed to determine their effects on the OHIP-14 domains. Table 5 shows these results. The effects of study times and groups were also included.

In general terms, only the variable group (treatment type) has a significant effect on the evolution of OHIP-14. Regression models show some isolated effects of individual variables on some of the domains.

IOTN shows statistically significant results in the total score and in 4 of the domains. When analyzing the coefficients of the model, it can be observed that the higher the IOTN score, the higher the OHIP-14 scores.

Marital status shows significant results for the handicap domain. The coefficients of the model show that married patients obtained lower scores than single patients for this domain.

The rest of the studied variables did not significantly influence the OHIP-14 scores.

## 4. Discussion

In recent years, the concept of health, and the biomedical and biopsychosocial models on which it is based, have undergone a paradigmatic shift, whereby new concepts such as health-related quality of life and, in the field of dentistry, oral health-related quality of life have grown in importance [16]. In this context, the objective of the present study was to evaluate the changes in oral health-related quality of life experienced by orthodontic patients by comparing four types of treatment by means of the OHIP-14 questionnaire.

The first part of the work consisted of analyzing the total scores by simply totaling the scores obtained for the 14 questions that make up the OHIP-14 questionnaire. Secondly, each of the questionnaire’s seven domains or partial components were analyzed separately: functional limitation, physical pain, psychological discomfort, physical disability, psychological disability, social disability, and handicap.

This is the first study that has applied a range of statistical methods to assess changes in quality of life scores for four types of treatment (buccal metal brackets, buccal esthetic/ceramic brackets, lingual brackets, and clear aligners) over three study times (pre-treatment, 6 months after the start of treatment, at the end of treatment).

As in the present work, other authors [6,9,10] have also evaluated the evolution of quality of life over time, applying questionnaires at four or six study times, or pre-treatment and post-treatment, or after 2 years of treatment. However, these authors only evaluated a single type of orthodontic treatment with conventional brackets. Two studies [17,18] compared invisible orthodontics with conventional brackets but only over fixed study periods. A single comparison between initial and final evaluations is inadequate as the patients comprising the study groups may present different baseline conditions. AlSeraidi et al. assessed the differences in OHRQL of patients wearing 3 different orthodontic appliances only during the initial stages of treatment [19].

- General Evaluation

Regarding the initial assessment of patients’ malocclusions by means of the IOTN, the study obtained very homogenous data across the patient sample. However, it was found that at the pre-treatment study time, patients in the metal bracket group produced lower OHIP-14 scores, thus a better baseline quality of life. The other three groups presented a poorer quality of life, particularly the clear aligner (C) group. These differences at baseline could be due to the patient’s rigorousness: patients seeking esthetic orthodontic appliances, clear aligners in particular, are usually more demanding, both esthetically and functionally, than patients requesting metal appliances. After 6 months of treatment (T1), patients treated with buccal brackets, regardless of whether they were metal or esthetic/ceramic, presented the poorest quality of life, while the clear aligners (C) group showed almost no change in quality of life.

At the end of treatment, the overall situation presented high homogeneity between groups. Quality of life clearly improved in all groups, with very little variation within each group.

Some authors [10,11,20,21,22] also concluded that patients experienced an improvement in quality of life at the end of treatment with metal brackets. As in the present study, quality of life during the active treatment period decreased significantly but temporarily [10,11,21].

Other authors such as Zhang et al. [23] and Johal et al. [24] reported significant reductions in quality of life, especially during the first three months of treatment. At six months they obtained values similar to those recorded before the start of treatment. The study by Johal et al. [24] also obtained an increase in quality of life by the end of orthodontic treatment. AlSeraidi et al. assessed OHRQL at one treatment stage (6–9 weeks after the beginning of treatment) and found the highest quality of life for patients wearing aligners. When comparing these results to our T1 results, similar results can be observed, although different questionnaires were used [19].

- Evaluation of Individual OHIP-14 Domains

The results obtained for the domains’ functional limitation, physical pain, and psychological discomfort showed that patients in the lingual brackets (L) and clear aligners (C) groups presented lower pre-treatment quality of life levels than the two buccal brackets groups (M and E), especially those patients treated with clear aligners (C). After 6 months of active treatment, the values obtained for functional limitation worsened, but changes in physical pain and psychological discomfort did not reach statistical significance in the lingual orthodontics group (L), and in the clear aligners (C) group, physical pain actually decreased signifying an improvement in quality of life for this domain. At the end of treatment, the values obtained pointed to greater homogeneity between the groups, with a generalized improvement, although the metal brackets group (M) obtained better results for the physical pain domain.

Data obtained for the disability domains (physical disability, psychological disability, social disability, and handicap) showed that patients’ baseline quality of life was better in the clear aligners group (C), while patients treated with brackets showed the poorest pre-treatment results for general handicap. After 6 months of active treatment, patients treated with clear aligners presented improvement or stability for these domains, while the other groups presented the typical pattern of short medium-term reductions in quality of life followed by notable improvement by the end of treatment.

The reduction in quality of life during treatment was greater among patients treated with brackets. At the end of treatment, all groups showed general improvements in these domains, but the invisible orthodontics group showed less improvement as quality of life for this group had not worsened during treatment to such an extent.

Authors such as Zhang et al. [23] and Cheng et al. [10] also reported significant changes in the domains’ functional limitation, physical pain, psychological discomfort or physical disability among patients treated with brackets, particularly during the first few weeks of active treatment. Zhang et al. [23] did not find statistically significant changes in social disability during treatment with brackets. Flores-Mir et al. [17] found that all patients underwent similar improvements in quality of life by the end of treatment.

Regarding gender, only isolated differences were found in the present study in the physical pain and physical disability domains, females scoring significantly higher than males. Some of the studies that analyze oral health-related quality of life during orthodontic treatment have also assessed gender differences. Our results agree with those found by Benson et al. [7] who did not find significant associations between the gender of the participant and the OHRQL. Two studies assessing self-esteem (SE) during orthodontic treatment found contradictory results regarding gender. While Jung found an increase in SE in males compared to females, Vontrood et al. reported the opposite [25,26].

The present study analyzed the potential influence of individual factors such as marital status, educational level, gender, age, and IOTN in the OHRQL. IOTN was found to influence some of the domains, scoring higher when the IOTN was also high. These results are in agreement with the systematic review conducted by Sun et al. [27]. Regarding age, marital status, and educational level, no significant differences were detected. Only one isolated association on the marital status variable was found for one domain. Miller et al. found age to be a significant factor with more negative impact and pain associated with younger ages. They also studied the educational-level factor but did not find any interactions as in the present study [18]. AlSeraidi et al. collected the same patient-related data as our study but did not analyze the associations with OHRQL scores [19].

Patients with malocclusions can develop feelings of low self-esteem, or embarrassment derived from their oral health status, which impact negatively on their general quality of life [28]. In this way, many patients seek orthodontic treatment in the hope of improving not only oral function but also dental esthetics and smile esthetics [29], which will in turn boost their self-esteem [9]. As the present study shows, the use of the OHIP-14, already shown to be a reliable and valid instrument for measuring quality of life [10,11], provides useful information about changes in the different physical, psychological, and social components that constitute oral health-related quality of life. The fact that the quality of life among patients treated with clear aligners did not worsen significantly during treatment, and even improved in some OHIP-14 domains, could be due to the innate characteristics of the appliance used, particularly its transparency or ‘invisibility.’ These characteristics would appear to help patients enjoy higher self-esteem, confidence, and self-assurance during treatment. While it is clear that all the groups treated enjoyed a good quality of life as a result of treatment, quality of life during treatment is also a factor worthy of consideration.

To date, no studies have provided quality of life data for four different types of orthodontic treatment. As stated above, two studies [17,18] have compared quality of life between invisible orthodontics and brackets but only during the first week of treatment compared with the end of treatment, and another study [19] assessed the OHRQL of three different appliances only at one treatment stage (6–9 weeks after the beginning of treatment) thus not analyzing changes. For this reason, the present findings should be treated with caution as more studies with comparable protocols are needed to confirm the results and gain a clearer picture of the evolution of OHRQL during different types of orthodontic treatment.

## 5. Conclusions

- In general, patients underwent a significant reduction in quality of life during treatment in comparison with their pre-treatment condition but enjoyed significant improvements at the end of treatment.
- The short medium-term (6 months) negative impact of orthodontic treatment on quality of life was greater among patients treated with buccal brackets. In the lingual brackets group, psychological discomfort and general handicap did not worsen during treatment. In the clear aligners group, physical and psychological disability did not worsen during treatment.
- Regarding overall changes in quality of life from pre-treatment to post-treatment, almost all the OHIP-14 domains underwent improvements in all groups. The only exceptions were that functional limitation remained unchanged in the metal brackets group, as did physical pain suffered by patients treated with esthetic/ceramic brackets and lingual orthodontics.

## Figures and Tables

**Table 1:** Homogeneity of the groups of treatment according to total OHIP-14 scores in T0: Kruskal–Wallis test results.

	*p*-Value
Q1. Problems with pronunciation	<0.001 ***
Q2. Bad sense of taste	<0.001 ***
Q3. Pain	<0.001 ***
Q4. Discomfort when eating	<0.001 ***
Q5. Concern for the mouth	0.046 *
Q6. Self-consciousness	<0.001 ***
Q7. Dissatisfaction with food intake	0.008 **
Q8. Interruption of meals	0.328
Q9. Dificulty in relaxing	0.120
Q10. Embarrassement	<0.001 ***
Q11. Irritability	0.071
Q12. Problems at work	0.016 *
Q13. Less satisfying life	0.003 **
Q14. Complete inability to function	<0.001 ***

* *p* < 0.05; ** *p* < 0.01; *** *p* < 0.001.

**Table 2:** Evolution of OHIP-14 total score of each group: median (range) and test ATS results with Brunner–Langer model, Kruskal–Wallis test and Mann–Whitney test with Bonferroni correction for comparisons between groups.

	T0	T1	T2	TIME	T0 vs. T1	T1 vs. T2	T0 vs. T2	*p*-Value
Metal brackets (M)	11.5 (7–17)	24.0 (19–31)	2.0 (0–5)	<0.001 ***	<0.001 ***	<0.001 ***	<0.001 ***	Time *p* < 0.001 *** Group *p* = 0.023 * Interaction *p* < 0.001 ***
Esthetic Brackets (E)	13.0 (8–22)	25.0 (18–30)	2.0 (1–6)	<0.001 ***	<0.001 ***	<0.001 ***	<0.001 ***	
Lingual Brackets (L)	12.0 (6–24)	21.0 (13–27)	2.0 (0–5)	<0.001 ***	<0.001 ***	<0.001 ***	<0.001 ***	
Clear aligners (C)	13.5 (10–25)	15.0 (10–21)	3.0 (1–6)	<0.001 ***	0.003 **	<0.001 ***	<0.001 ***	
GROUP	0.013 *	<0.001 ***	<0.001 ***					
M vs. E	0.006 **	1.000	1.000					
M vs. L	1.000	0.018 *	1.000					
M vs. C	0.018 *	<0.001 ***	<0.001 ***					
E vs. L	1.000	0.006 **	1.000					
E vs. C	1.000	<0.001 ***	0.036 *					
L vs. C	0.924	<0.001 ***	<0.001 ***					

* *p* < 0.05; ** *p* < 0.01; *** *p* < 0.001.

**Table 3:** Homogeneity of the groups of treatment according with partial and total OHIP-14 scores in T0: Kruskal–Wallis test results.

	*p*-Value
Q1 + Q2. Functional Limitation	<0.001 ***
Q3 + Q4. Physical pain	<0.001 ***
Q5 + Q6. Psychological discomfort	<0.001 ***
Q7 + Q8. Physical disability	0.011 *
Q9 + Q10. Psychological disability	<0.001 ***
Q11 + Q12. Social disability	0.002 **
Q13 + Q14. Handicap	<0.001 ***
OHIP-14 Total score	0.013 *

* *p* < 0.05; ** *p* < 0.01; *** *p* < 0.001.

**Table 4:** Comparison between means in OHIP-14 scores for the four types of orthodontic treatment at three study times (T0, T1, and T2).

OHIP-14 Domains	Study Time	Total	Male	Female	*p*-Value < 0.05
Functional limitation	T0	1.22	1.21	1.22	
	T1	2.12	2.15	2.08	
	T2	0.39	0.39	0.39	
Physical pain	T0	1.21	1.20	1.22	
	T1	3.29	3.43	3.15	
	T2	0.73	0.59	0.88	*
Psychological discomfort	T0	2.31	2.20	2.42	
	T1	3.10	3.07	3.14	
	T2	0.29	0.25	0.34	
Physical disability	T0	0.61	0.62	0.59	
	T1	3.09	3.23	2.95	
	T2	0.18	0.08	0.29	*
Psychological disability	T0	3.13	3.20	3.05	
	T1	3.71	3.80	3.61	
	T2	0.23	0.23	0.22	
Social disability	T0	2.42	2.43	2.41	
	T1	3.13	3.25	3.02	
	T2	0.42	0.49	0.34	
Handicap	T0	2.26	2.26	2.25	
	T1	2.57	2.52	2.61	
	T2	0.35	0.34	0.36	
Total score	T0	13.14	13.11	13.17	
	T1	21.01	21.44	20.56	
	T2	2.59	2.38	2.81	

* *p*-value < 0.05.

**Table 5:** Effects of groups, study times, and patient-related variables on OHIP-14 domains. Multiple regression models with generalized estimation equations (GEE) results.

OHIP-14 Domains	Study Time	Group	Study Time/Group	Gender	Age	Marital Status	Educational Level	IOTN
Functional limitation	<0.001 ***	<0.001 ***	<0.001 ***	0.160	0.169	0.069	0.310	<0.001 ***
Physical pain	<0.001 ***	<0.001 ***	<0.001 ***	0.827	0.341	0.455	0.788	<0.001 ***
Psychological discomfort	<0.001 ***	0.004 **	<0.001 ***	0.465	0.909	0.464	0.260	<0.001 ***
Physical disability	0.011 *	<0.001 ***	<0.001 ***	0.709	0.118	0.150	0.061	0.138
Psychological disability	<0.001 ***	<0.001 ***	<0.001 ***	0.181	0.775	0.394	0.477	<0.001 ***
Social disability	0.002 **	0.001 **	0.017 *	0.124	0.436	0.096	0.101	0.191
Handicap	<0.001 ***	<0.001 ***	<0.001 ***	0.284	0.149	0.033 *	0.552	0.065
OHIP-14 Total score	<0.001 ***	<0.001 ***	<0.001 ***	0.322	0.243	0.382	0.561	<0.001 ***

* *p* < 0.05; ** *p* < 0.01; *** *p* < 0.001.

## Data Availability

The data presented in this study are available on request from the corresponding author.
